# Emerging roles of apolipoprotein E4 in cerebral small vessel disease-related cognitive impairment: mechanistic insights and therapeutic perspectives

**DOI:** 10.3389/fnhum.2026.1957032

**Published:** 2026-10-13

**Authors:** Mengru Li, Yu Zhang, Peizhu Lü, Junchen Li, Yu Zhang, Shun Wang, Yan Bai, Yulin Wang

**Affiliations:** 1 Heilongjiang University of Chinese Medicine, Harbin, Heilongjiang, China; 2 Heilongjiang University of Chinese Medicine Affiliated Hospital, Harbin, Heilongjiang, China

**Keywords:** apolipoprotein E4, blood–brain barrier, cerebral small vessel disease, lipid homeostasis, neuroinflammation, neurovascular unit, vascular cognitive impairment

## Abstract

Cerebral small vessel disease (CSVD) is a major contributor to cognitive decline and vascular cognitive impairment in the elderly. However, the extent to which apolipoprotein E4 (ApoE4) contributes to sporadic, non-amyloid CSVD remains unclear. Previous literature has often inferred CSVD mechanisms from Alzheimer’s disease (AD) research, carrying the risk of overstating disease-specific causal relationships. Therefore, this narrative review re-evaluates the relationship between ApoE4 and CSVD using a CSVD-centric hierarchy of evidence. Population-based imaging and epidemiological data suggest that “APOE” ε4 may alter susceptibility to white matter injury and cognitive impairment; however, the effect sizes are generally small or context-dependent, and associations are influenced by factors such as age, hypertension, diabetes, total vascular risk burden, AD pathology, ancestry, sex, and “APOE” gene dosage. Evidence is stronger for cerebral amyloid angiopathy (CAA)—which involves mechanisms distinct from sporadic arteriolosclerotic CSVD—and thus warrants separate discussion. At the mechanistic level, direct evidence regarding CSVD supports endothelial dysfunction, BBB leakage, neurovascular unit (NVU) instability, and white matter injury as core pathological processes. Among the evidence specific to ApoE4, models involving impaired neurovascular regulation and chronic hypoperfusion are the most compelling; conversely, mechanisms such as the cyclophilin A–MMP-9 pathway, TREM2–ApoE signaling, insulin receptor trafficking, reelin-related synaptic effects, endoplasmic reticulum stress, and autophagy currently rely primarily on AD research or preclinical evidence. Two recently proposed specific hypotheses—the brain–heart–kidney axis hypothesis and the microglial lipid droplet model—offer valuable conceptual frameworks but should be viewed as testable theories rather than established mechanisms. To date, no ApoE4-targeted therapies have been proven effective for CSVD-related cognitive impairment future research should prioritize genotype-stratified longitudinal CSVD cohorts, standardized imaging studies, adjustment for Alzheimer’s disease (AD) biomarkers, analysis of interactions between vascular risk factors, studies on human white matter tissue, and the development of ApoE4 disease models that integrate chronic hypoperfusion and vascular comorbidities.

## 1 Introduction

Cerebral small vessel disease (CSVD) encompasses a range of pathological processes affecting small arteries, arterioles, capillaries, and venules; on magnetic resonance imaging (MRI), it manifests as features such as white matter hyperintensities (WMH), lacunes, cerebral microbleeds (CMBs), and enlarged perivascular spaces (EPVS) (Wardlaw et al., 2019). CSVD is highly prevalent among the elderly; recent systematic reviews and meta-analyses indicate that MRI-defined small vessel disease affects approximately one-fifth of adults overall, with prevalence increasing with age (Gao et al., 2022). Among 3,063 community-dwelling adults aged 50–75 in China, 30.5% exhibited at least one major CSVD imaging marker, with the respective prevalences of confluent WMH, CMB, moderate-to-severe basal ganglia EPVS, and lacunes being 16.7%, 10.2%, 9.8%, and 5.6% (Yang Y. et al., 2023).

CSVD serves as a key pathological basis for vascular cognitive impairment (VCI), often manifesting as deficits in executive function, attention, and information processing speed (Gao et al., 2022; Sweeney et al., 2019). In this review, CSVD-CI refers to cognitive impairment primarily attributable to CSVD, representing an etiological subtype within the broader VCI spectrum (Litak et al., 2020; Zanon Zotin et al., 2021). While WMH burden is associated with cognitive impairment and the risk of dementia, CSVD-CI is characterized by significant heterogeneity and frequently co-occurs with neurodegenerative pathology (Wardlaw et al., 2019; Sweeney et al., 2019). APOE ε4 is a significant genetic risk allele for sporadic Alzheimer’s disease (AD) (Mahley, 2016; Jackson et al., 2024), and ApoE4 is the protein isoform it encodes; “unlike established monogenic CSVD genes such as NOTCH3 and COL4A1/2, APOE ε4 is currently regarded as a susceptibility factor rather than an established monogenic cause of sporadic CSVD” (Schilling et al., 2013; Luo et al., 2017; Whittaker et al., 2022).

Existing research suggests that ApoE4 may alter the susceptibility of brain tissue to vascular injury and neurodegenerative changes by influencing neurovascular unit (NVU) homeostasis, BBB integrity, inflammatory regulation, and cerebral lipid metabolism (Liu et al., 2022;Liu et al., 2023; Montagne et al., 2020; De Leeuw et al., 2022). However, most mechanistic evidence stems from AD-related studies, humanized animal models, and cell-based experiments, with limited direct validation in the context of sporadic CSVD. Therefore, it is more appropriate to regard ApoE4 as a susceptibility-modifying factor that interacts with traditional vascular risk factors, rather than as a confirmed causative gene for CSVD.

Accordingly, this review aims to: distinguish evidence specific to CSVD from inferences derived from AD; discuss sporadic arteriolosclerotic CSVD separately from cerebral amyloid angiopathy (CAA); and avoid characterizing unverified cross-disease pathways as established causes of CSVD-CI when integrating vascular, metabolic, and inflammatory mechanisms. Recently proposed theoretical models specific to CSVD are also discussed, with the level of evidence for each clearly indicated.

## 2 Literature search strategy

This study is a narrative review employing a structured literature search method. We searched PubMed and Web of Science databases for literature published up to September 1, 2026, using “APOE4” or “apolipoprotein E4” in combination with keywords such as “cerebral small vessel disease,” “white matter hyperintensity,” “cerebral microbleed,” “cerebral amyloid angiopathy,” “blood–brain barrier,” “neurovascular unit,” “endothelial,” “pericyte,” “microglia,” “lipid droplet,” “cholesterol,” “vascular cognitive impairment,” and “cognition.” References from relevant systematic reviews and original studies were also traced. After removing duplicates, search results were screened based on titles, abstracts, and full texts. Priority was given to epidemiological and neuroimaging studies involving CSVD populations, as well as recent disease-related research; however, earlier seminal papers were retained where necessary to describe established, classic mechanisms. Studies on Alzheimer’s disease (AD), cells, or animals were included only if they involved mechanistic pathways potentially relevant to CSVD and were explicitly identified as indirect evidence. Reports unrelated to cerebrovascular or cognitive outcomes, duplicate publications, and mechanistic assertions lacking traceable original literature sources were not prioritized.

To avoid overinterpretation, this article discusses mechanisms using a three-tier hierarchy of evidence. Tier 1 refers to direct evidence—epidemiological, imaging, or histological—derived from clearly defined CSVD populations. Tier 2 refers to suggestive evidence derived from disease-related experimental models (e.g., vascular stress or chronic hypoperfusion mimicking CSVD) or human studies involving CSVD patients who also present with significant competing pathologies. Tier 3 refers to indirect or speculative evidence derived primarily from AD, other neurodegenerative diseases, or “*in vitro*” systems, which has not yet been directly confirmed in the context of CSVD. The terms “established,” “suggestive,” and “speculative” in Table 1 specifically denote the strength of evidence regarding the pathway’s role in the ApoE4–CSVD relationship, rather than the sufficiency of biological evidence for that pathway in other diseases.

**Table 1:** Evidence-strength assessment of proposed ApoE4-related mechanisms in CSVD-CI.

Mechanism	Cellular locus/mediators	Direct CSVD evidence	Evidence strength for ApoE4-CSVD link	Interpretation
Endothelial/NVU dysfunction	Endothelium; eNOS/NO; neurovascular coupling	Strong evidence that endothelial/NVU dysfunction is central to CSVD; ApoE4-specific human data limited	Suggestive	Plausible susceptibility pathway; distinguish CSVD pathology from ApoE4-specific causation
BBB/pericyte injury	Endothelium/pericytes; BBB leakage; CypA-MMP-9	BBB leakage is established in CSVD; CypA-MMP-9 evidence is mainly ApoE4/AD-related	Suggestive for BBB; speculative for CypA-MMP-9 in CSVD	Do not present CypA-MMP-9 as an established sporadic-CSVD mechanism
Microglial lipid droplets	Microglia; ACSL1; lipid droplets; lipid peroxidation	2025 CSVD-specific hypothesis; direct longitudinal/pathological validation lacking	Hypothesis/suggestive	High relevance to WMH but currently unvalidated (Luo and Qiao, 2026)
TREM2-ApoE microglial program	Microglia; TREM2-ApoE; NF-κB	No direct independent CSVD demonstration	Speculative	Predominantly AD-derived
Cholesterol transport/lipidation	Astrocytes, microglia; ABCA1; LDLR/LRP1	CSVD relevance biologically plausible; ApoE4-specific molecular evidence mainly AD/cell-based	Speculative to suggestive	Potential modifier of myelin/NVU resilience; disease-specific causality unproven
Glucose/insulin signaling	Neurons/glia; insulin receptor; PI3K-Akt; mitochondria	No direct ApoE4-CSVD demonstration	Speculative	AD-derived mechanism that may affect tolerance to chronic hypoperfusion
ER stress/autophagy	Neurons/glia; PERK/IRE1/ATF6; mTOR/ULK1; lysosome	No direct ApoE4-CSVD demonstration	Speculative	Retained as testable cellular-stress pathway
Synaptic/Reelin-ApoER2 signaling	Neurons; ApoER2; Reelin-Dab1; NMDA/AMPA	No direct ApoE4-CSVD demonstration	Speculative	Final common neuronal mechanism, not a proven CSVD-specific driver

Ratings refer specifically to evidence that the ApoE4-linked pathway operates in CSVD, not to whether the pathway is established in AD or other diseases.

## 3 Structure and function of apolipoprotein E

ApoE is a lipid transport protein composed of 299 amino acids; its three common isoforms are encoded by the APOE ε2, ε3, and ε4 alleles, respectively. ApoE is involved in lipoprotein assembly, receptor-mediated lipid transport, and the maintenance and repair of cell membranes (Mahley, 2016; Mehta and Shapiro, 2022; Windham and Cohen, 2024). Compared with ApoE2 and ApoE3, ApoE4 exhibits a specific tendency for domain interaction due to the presence of arginine at position 112 (Arg112); this conformational change may affect protein stability and lipid-binding properties (Sun et al., 2023). Specifically, Arg112 alters the conformation of the N-terminal domain and reorients the side chain of arginine at position 61 (R61), facilitating its interaction with glutamate at position 255 (E255) in the C-terminal domain and contributing to the more compact conformation of ApoE4 compared with ApoE3 (Ray et al., 2017; Moon et al., 2024). This conformational difference may influence the efficiency of ApoE4-mediated lipoprotein assembly, intracellular transport, and lipid delivery, thereby affecting lysosomal-autophagic function, mitochondrial homeostasis, and inflammatory responses (Yang L. G. et al., 2023). Current insights into these mechanisms are primarily derived from models of neurodegenerative diseases, and their specific roles in CSVD-related cognitive impairment require further validation (Windham and Cohen, 2024; Asiamah et al., 2024; Dias et al., 2025) (Figure 1).

**Figure 1:**
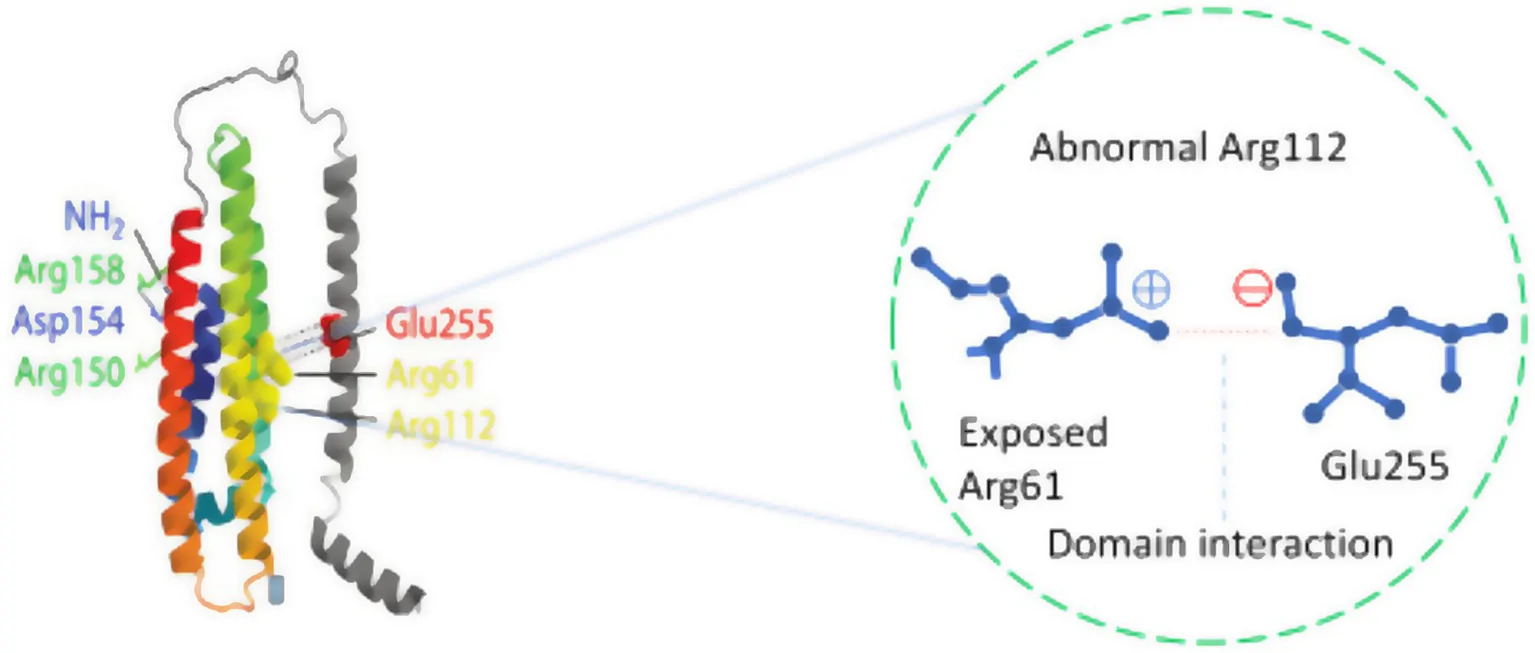
Structural characteristics of ApoE4 and the domain interaction underlying its altered biological properties. ApoE4-specific Arg112 promotes exposure of Arg61 and interaction with Glu255, producing a more compact conformation than ApoE3. This structural feature can influence protein stability, lipid binding, receptor interaction, and intracellular trafficking; its direct contribution to CSVD-related cognitive impairment remains unproven.

In the central nervous system, ApoE is primarily produced by astrocytes under physiological conditions, although microglia and neurons can also upregulate ApoE expression in response to stress (Lin and Holtzman, 2024). ABCA1-dependent lipidation is crucial for the formation and function of ApoE-containing lipoprotein particles (Windham and Cohen, 2024; Lindner et al., 2022). The structural differences associated with ApoE4 can influence lipid binding, intracellular transport, inflammatory responses, and cellular stress; however, much of the detailed molecular evidence regarding these effects stems from research models related to Alzheimer’s disease (AD) (Windham and Cohen, 2024; Blumenfeld et al., 2024). Further confirmation is required regarding the specific implications of these mechanisms in the context of CSVD. Regarding CSVD, the critical question is not merely whether ApoE4 exerts biological effects, but rather which of these effects can be confirmed to actually occur within the affected small vessels and white matter. Therefore, a CSVD-centric framework should begin with established disease processes—including endothelial dysfunction, impaired cerebral blood flow regulation, BBB leakage, chronic vascular inflammation, and white matter injury—and then examine whether APOE ε4 alters the timing, severity, or consequences of these processes. This approach avoids the pitfall of automatically attributing every ApoE4 phenotype observed in Alzheimer’s disease (AD) to the mechanisms of CSVD.

The recently proposed “brain–heart–kidney axis hypothesis” situates CSVD within the broader framework of systemic small vessel disease, positing that the microcirculations of the brain, heart, and kidneys may share common vascular, inflammatory, oxidative stress, and genetic mechanisms (Li et al., 2025a). As this 2025 paper presents a hypothesis rather than a validated new disease entity, it should be interpreted with caution; nevertheless, given that ApoE4 influences extracerebral lipid processing and vascular biology, the theory offers a useful organizing principle for understanding ApoE4. Within this framework, it is more appropriate to view ApoE4 as a susceptibility modifier whose impact may depend on the systemic vascular burden, rather than as an isolated, brain-specific pathogenic trigger. Validating this perspective requires the simultaneous characterization of cerebral CSVD, cardiac microvascular dysfunction, renal disease, and vascular risk factors in cohorts stratified by genotype.

Figure 2 summarizes this model, which incorporates a hierarchy of evidence. Solid lines indicate associations with the strongest current evidence; dashed lines represent CSVD-specific hypotheses requiring further validation; and dotted lines denote pathways supported primarily by data from AD or other indirect systems.

**Figure 2:**
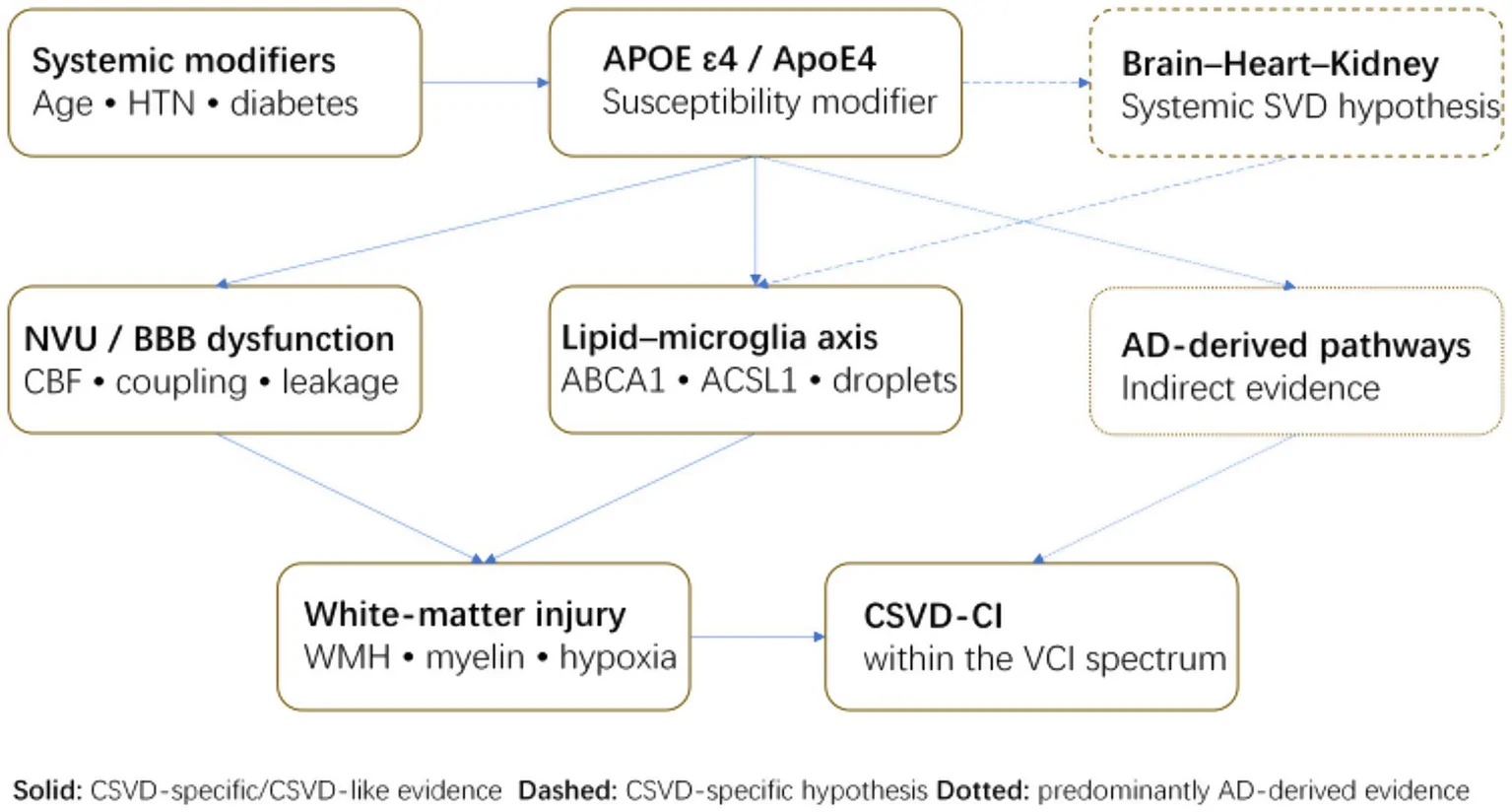
Evidence-aware, CSVD-centered conceptual framework linking APOE ε4/ApoE4 to CSVD-related cognitive impairment. ApoE4 is positioned as a susceptibility modifier acting in the context of age, hypertension, diabetes, and other systemic vascular risks. The Brain–Heart–Kidney axis hypothesis and the microglial lipid-droplet pathway are shown as testable CSVD-specific hypotheses, whereas pathways derived predominantly from Alzheimer’s disease are explicitly separated as indirect evidence. Solid, dashed, and dotted connections indicate progressively less direct support for the ApoE4–CSVD relationship, as defined in the main text.

## 4 Epidemiological and neuroimaging evidence

### 4.1 Sporadic arteriolosclerotic CSVD and white matter damage

Associations between APOE ε4 and sporadic CSVD vary across different populations and outcome measures. In the Rotterdam Scan Study, researchers assessed APOE genotypes, blood pressure, and white matter lesions in 971 participants. Compared with ε3/ε3 subjects, ε4 carriers exhibited greater subcortical white matter lesion volumes (adjusted mean difference: 0.5; 95% CI: 0.2–0.8); furthermore, participants with both hypertension and the ε4 allele showed the highest lesion burden, with a statistically significant interaction effect observed for subcortical lesions (*p* = 0.016, de Leeuw et al., 2004; Jin et al., 2025; Marini et al., 2020). This finding is significant because it supports a “gene–environment interaction” model rather than a model determined solely by genotype.

A longitudinal study based on the ADNI cohort, involving 135 cognitively normal older adults, found that ε4 carriers had a greater burden of frontal WMH and basal ganglia EPVS at baseline, as well as faster progression of frontal WMH (Luo et al., 2017; Lyall et al., 2020). However, as the cohort included cerebrospinal fluid and amyloid-PET markers—and associations with amyloid-beta (Aβ) were observed—it is difficult to attribute these associations entirely to non-amyloid, arteriolosclerotic CSVD. Consequently, while the study offers valuable insights, it cannot be regarded as “pure” evidence regarding hypertensive CSVD.

More recent pooled evidence further highlights this inconsistency. A 2024 meta-analysis comprising 12 studies and 16,738 adults found no statistically significant overall difference in WMH volume between APOE ε4 carriers and non-carriers (Hedges’ *g* = 0.07; 95% CI: −0.01–0.15; *p* = 0.09); only a modest association was observed in community-based populations (*g* = 0.09; 95% CI: 0.02–0.16; *p* = 0.008), whereas results from clinical cohorts were null (Stipho and Malek-Ahmadi, 2024). A 2025 NEUROGEN-SVD meta-analysis indicated that APOE ε4 carriage is associated with global cognitive impairment in CSVD study populations (OR 1.80, 95% CI 1.41–2.29); however, there was high heterogeneity across studies, and the certainty of evidence was low (Jin et al., 2025). More notably, recent studies specifically enrolling CSVD patients have yielded results showing greater phenotypic specificity: Jin et al. found that in 717 CSVD patients, APOE ε4 gene dosage showed a dose-dependent relationship with cognitive decline and WMH severity, and was associated with elevated LDL-C (Jin et al., 2026); meanwhile, Liu and Gou found that in 728 patients with chronic lacunar infarction, APOE ε4 was independently associated only with periventricular WMH burden—showing no significant association with global CSVD burden, deep WMH, cerebral microbleeds (CMBs), or EPVS—while smoking and alcohol consumption were found to modify certain CSVD phenotypes (Liu and Gou, 2026). Collectively, these studies suggest that the effects of APOE ε4 may be dose-dependent, imaging-phenotype specific, and environmentally dependent, rather than exerting a uniform effect across the entire CSVD spectrum.

Interpretation of these results must also account for gene dosage and population structure. ε4/ε4 homozygotes are not necessarily equivalent to heterozygous carriers, and APOE allele frequencies and linkage patterns vary across populations of different ancestries. Factors such as age, sex, ancestry, education level, hypertension, diabetes, dyslipidemia, smoking, renal disease, overall vascular risk, and co-existing Alzheimer’s disease (AD) pathology may all alter the true biological effects or the observed statistical associations. Therefore, future studies should report ε4 gene dosage, ancestry, education level, vascular risk factor exposure, and AD biomarkers, rather than relying solely on a “carrier/non-carrier” classification for analysis.

### 4.2 Cerebral amyloid angiopathy should not be conflated with sporadic CSVD

Cerebral amyloid angiopathy (CAA) is characterized by the deposition of amyloid-β protein in cortical and leptomeningeal vessels and is closely associated with lobar CMBs and intracerebral hemorrhage. Although CAA is often included within the broad spectrum of small vessel disease, its underlying mechanisms differ fundamentally from the lipohyalinosis and arteriolosclerosis driven by hypertension, aging, and other vascular risk factors. APOE ε4 promotes cerebral amyloid deposition and has a clear biological link to amyloid-related vascular pathology; therefore, findings from CAA studies cannot be directly cited as evidence that ApoE4 causes sporadic hypertensive CSVD.

Consequently, this review treats research on lobar WMH and CAA as evidence of an “amyloid-related small vessel phenotype” rather than proof of a link between ApoE4 and sporadic CSVD. Whenever feasible, deep CMBs, lacunes, basal ganglia EPVS, and non-amyloid WMH should be analyzed separately. If studies combine these outcomes into a single CSVD score, they should report whether the results persist after excluding potential CAA cases and adjusting for amyloid biomarkers.

### 4.3 Summary of epidemiological evidence

Table 2 summarizes representative studies addressing the ApoE4–CSVD relationship, detailing study populations, APOE comparison methods, CSVD outcomes, effect estimates, and key confounders. The overall pattern does not indicate a consistent causal relationship: early studies supported an interaction between vascular risk factors and APOE ε4; longitudinal studies and recent research on CSVD patients suggest associations with WMH or cognition, though effects show marked phenotypic and cohort heterogeneity; pooled effects for WMH are small and inconsistent; and while cognitive associations reached statistical significance in meta-analyses, the certainty of the evidence remains low. Consequently, current evidence better supports characterizing ApoE4 as a susceptibility modifier—dependent on context, gene dosage, and vascular exposure—rather than a universal, direct causative factor for sporadic CSVD.

**Table 2:** Key human evidence for the association between APOE ε4/ApoE4 and CSVD-related phenotypes.

Study/design	Sample and APOE groups	CSVD-related outcome	Effect/principal result	Key adjustment or caveat	Evidence relevance
Luo et al. (2017); 2-year longitudinal ADNI cohort	*n* = 135 cognitively healthy older adults; ε2 carriers, ε4 carriers, ε3/ε3	WMH, dPVS, lacunes, CMBs, cognition	ε4 carriers showed more frontal WMH and faster frontal WMH progression; APOE ε4 × frontal WMH predicted MMSE change (*p* ≈ 0.05)	Age, sex, hypertension score, smoking, diabetes, CSVD markers; WMH group effect was attenuated and disappeared when CSF Aβ1-42 was included	Human longitudinal evidence, but AD biomarkers/ADNI context limit attribution to pure non-amyloid CSVD (Luo et al., 2017)
Lyall et al. (2020); UK Biobank cross-sectional imaging	*n* = 8,395; ε4 carriers vs. noncarriers	WMH volume and white-matter integrity	Higher WMH volume in ε4 carriers: standardized *β* = 0.088 (95% CI 0.036–0.139), *p* = 0.001; no global FA/MD association	Adjusted for age, sex, deprivation, smoking, and cardiometabolic disease	Large human population study; supports a small association with WMH, not a uniform white-matter effect (Stipho and Malek-Ahmadi, 2024)
Stipho and Malek-Ahmadi (2024); meta-analysis of 12 studies	*n* = 16,738; 4,721 ε4 carriers, 12,017 noncarriers	WMH volume	Overall: Hedges *g* = 0.07 (95% CI -0.01 to 0.15), *p* = 0.09; community studies *g* = 0.09 (0.02–0.16), *p* = 0.008; clinic studies null	Heterogeneity in study setting and WMH segmentation; pooled analysis cannot fully separate AD/vascular confounding	Best summary of conflicting human WMH evidence; overall association not significant (Lyall et al., 2020)
Montagne et al. (2020); human DCE-MRI/CSF study	Cognitively normal APOE ε4 carriers and noncarriers	BBB permeability/pericyte injury	APOE ε4 carriers showed BBB breakdown associated with cognitive decline risk	Cohort was designed around ApoE4/AD risk rather than clinically defined sporadic CSVD	Human vascular evidence, but indirect, for sporadic CSVD (Montagne et al., 2020)
CAA/lobar CMB evidence	APOE ε4/CAA-related populations	Lobar CMBs and amyloid angiopathy	Association is strongest for lobar hemorrhagic markers in CAA	CAA has amyloid-dependent pathology distinct from hypertensive/arteriolosclerotic CSVD	CAA-specific; should not be generalized to sporadic non-amyloid CSVD (Jackson et al., 2024)

The table emphasizes sample size, genotype grouping, effect magnitude, important confounders, and whether evidence is directly informative for sporadic CSVD.

## 5 Mechanisms organized by strength of CSVD evidence

### 5.1 Endothelial dysfunction, neurovascular coupling, hypoperfusion, and BBB integrity

The development of CSVD-CI involves microvascular injury, BBB dysfunction, neuroinflammation, white matter damage, and disrupted neurovascular coupling. Endothelial dysfunction and reduced vascular reactivity are well-established pathological features of CSVD, yet the specific contribution of ApoE4 to these processes remains uncertain. Therefore, the following section clearly distinguishes between established CSVD pathological processes and ApoE4-related mechanisms inferred primarily from Alzheimer’s disease (AD) research or experimental models (Figure 3).

**Figure 3:**
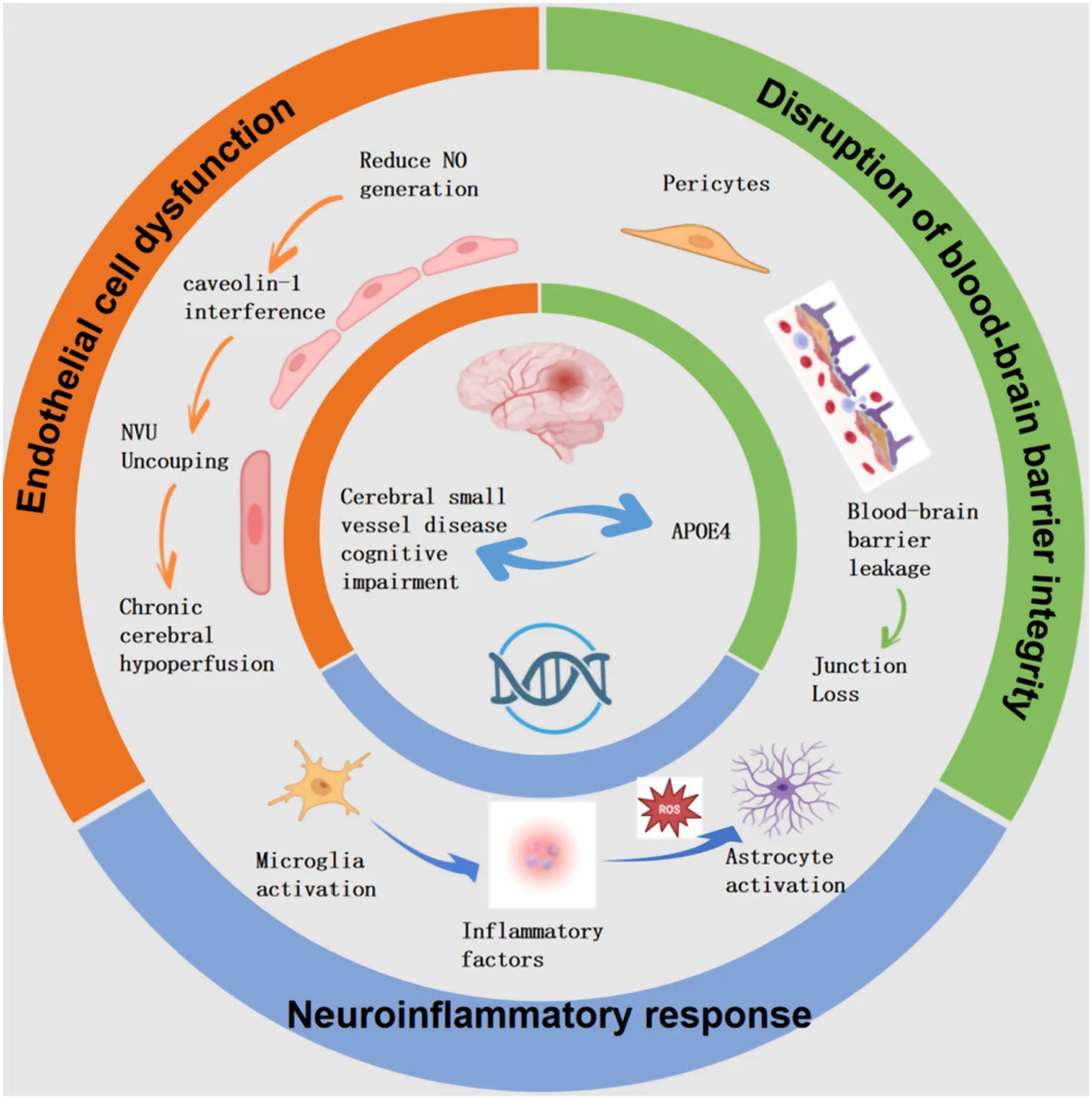
Proposed mechanisms linking ApoE4 with cerebrovascular dysfunction and cognitive impairment in CSVD. Endothelial dysfunction, BBB disruption, neuroinflammation, and NVU failure are established components of CSVD pathology, whereas most ApoE4-specific molecular links shown are supported by indirect or experimental evidence and require CSVD-specific validation.

Regardless of APOE genotype, endothelial dysfunction and BBB disruption are core components of CSVD (Gao et al., 2022; Jia et al., 2024; Sun and Liu, 2025). Recent CSVD-specific human imaging studies have further reinforced this foundation. In a study of 59 CSVD patients and 32 controls, dynamic contrast-enhanced MRI revealed spatially heterogeneous BBB leakage; a higher proportion of tissue within WMH exhibited leakage, and significant abnormalities were also observed in the “normal-appearing white matter” surrounding the lesions (Verstappen et al., 2025). Another longitudinal study involving 50 subjects with chronic cerebrovascular disease and WMH demonstrated that greater baseline BBB disruption predicted more pronounced subsequent WMH progression (Leigh et al., 2026). While these studies establish BBB dysfunction as a key component of the CSVD process, they do not in themselves prove that ApoE4 is the causative factor.

Evidence specific to ApoE4 is more compelling in disease-relevant vascular models. ApoE4-targeted replacement mice exhibit abnormalities in the cerebral blood flow and neurovascular regulation; under conditions of chronic cerebral hypoperfusion induced by bilateral common carotid artery stenosis, ApoE4—compared to ApoE3—exacerbates the reduction in white matter microvascular flow, local hypoxia, white matter injury, and cognitive dysfunction (Koizumi et al., 2018; Anfray et al., 2024). This represents some of the strongest mechanistic evidence linking ApoE4 to a CSVD-like phenotype, as the experimental stressor arises from vascular hypoperfusion rather than being driven by amyloid. Studies involving endothelial cells and iPSCs also suggest that different ApoE isoforms differentially affect endothelial junctions, the cytoskeleton, and intracellular trafficking (Koizumi et al., 2018); however, the extrapolation of these findings to human sporadic CSVD remains limited.

In contrast, the pericyte-based cyclophilin A (CypA)–MMP-9 pathway should be classified as indirect evidence. Cognitively normal APOE ε4 carriers can exhibit increased BBB permeability and elevated markers of pericyte injury, while AD-related models suggest the involvement of the WMH in ApoE4-associated BBB breakdown (Montagne et al., 2020; Bell et al., 2012). Although these findings provide a plausible molecular link, there is currently no direct evidence demonstrating that this pathway independently drives non-amyloid CSVD. Consequently, this should be framed as a testable hypothesis rather than an established mechanism of CSVD. Definitive experiments should involve interventions targeting CypA or MMP-9 in models of arteriolosclerotic or hypoperfusion-induced CSVD (comparing ApoE4 and ApoE3 genotypes) while simultaneously quantifying small-vessel pathology, BBB permeability, white matter injury, and cognitive outcomes.

### 5.2 Lipid homeostasis and the microglial lipid droplet hypothesis

Dysregulated lipid metabolism represents a biologically plausible link between ApoE4 and white matter vulnerability, as myelin maintenance, membrane repair, glial cell function, and vascular homeostasis all rely on tightly regulated lipid transport. ApoE-containing lipoproteins undergo lipidation via ABCA1 and deliver cholesterol and phospholipids to cells through pathways involving the LDL receptor family (Windham and Cohen, 2024; Yang L. G. et al., 2023; Husain et al., 2021). In both Alzheimer’s disease (AD) and cellular models, ApoE4 has been shown to alter lipoprotein composition, cholesterol efflux, intracellular transport, and lysosomal cholesterol processing (Yang L. G. et al., 2023; Husain et al., 2021; Liu et al., 2025). While these findings confirm the existence of ApoE isoform-specific lipid biology, they do not, in isolation, demonstrate a causal pathway by which ApoE4 leads to CSVD.

Microglia may represent a more disease-relevant intersection between lipid metabolism and white matter injury. Research in AD indicates that ApoE4 alters microglial lipid processing and promotes lipid droplet accumulation, accompanied by changes in inflammatory status, mitochondrial function, and phagocytic activity (Liu et al., 2023; Haney et al., 2024). Although the TREM2–ApoE transcriptional program plays a crucial role in neurodegenerative diseases (Li et al., 2022; Fitz et al., 2021), there is currently no direct evidence that this signaling axis independently drives CSVD; consequently, within the context of CSVD, this evidence is classified as Tier 3.

A 2025 article published in “Medical Hypotheses” proposed a CSVD-specific model suggesting that BBB dysfunction and systemic lipid exposure promote microglial lipid droplet accumulation, thereby contributing to the formation of WMH (Li et al., 2025b). This hypothesis also links lipid metabolism genes—such as ApoE4 and ACSL1—to the accelerated formation of lipid droplets. This hypothesis is directly relevant to the present review as it links ApoE4 to WMH—the most common MRI manifestation of CSVD (Haney et al., 2024; Li et al., 2025b). However, the nature of the evidence must be clearly understood: it is a mechanistic hypothesis synthesized from observations across genetics, lipid metabolism, microglia, and neurodegenerative diseases, rather than proof that “ApoE4-driven lipid droplets cause WMH in humans.”

Nevertheless, a testable sequence of events can be proposed: vascular injury and BBB leakage may increase local tissue exposure to circulating lipids; ApoE4-associated defects in lipid transport may impair adaptive lipid redistribution; ACSL1-mediated enhancement of fatty acid esterification may promote the storage of triglyceride-rich lipid droplets; oxidized lipids and lipid peroxidation products may amplify reactive oxygen species (ROS) and inflammatory signaling; and dysfunctional microglia may compromise support for oligodendrocytes and myelin, thereby promoting axonal and white matter injury. Candidate lipid species include triglycerides, cholesterol/cholesterol esters, phospholipids, and oxidized fatty acid products, though it remains unclear which class predominates in human CSVD lesions. Similarly, the temporal relationship—whether lipid droplet formation precedes, accompanies, or follows the development of WMH—has not yet been established. These represent critical gaps to be addressed by future research utilizing lipidomics, spatial transcriptomics, histology, and longitudinal imaging.

### 5.3 Neuroinflammation: an amplifier rather than an ApoE4-specific etiological factor

Chronic low-grade neuroinflammation is common in CSVD and can be driven by factors such as endothelial injury, BBB leakage, ischemia, oxidative stress, and glial cell activation (Sun and Liu, 2025; Luo and Qiao, 2026). Plasma proteins and inflammatory mediators entering the brain parenchyma can activate microglia and astrocytes, while cytokines and reactive oxygen species (ROS) can further damage the endothelium, oligodendrocytes, axons, and myelin. This creates a “vascular-glial” feed-forward amplification loop associated with CSVD, regardless of APOE genotype (Luo and Qiao, 2026).

ApoE4 may alter the activation threshold or cellular phenotype of this response. Experimental and Alzheimer’s disease (AD)-related studies have reported ApoE isoform-specific changes in microglial inflammatory signaling and altered responses to immune stimuli (Yin et al., 2023; Lee et al., 2023; Liu et al., 2024). However, findings from human brain tissue studies are not entirely consistent, and results are influenced by disease stage and cellular context. Therefore, a more appropriate interpretation is that ApoE4 may amplify or reshape neuroinflammation within a vascular environment already rendered vulnerable; however, direct evidence that ApoE4-specific inflammation initiates sporadic CSVD remains lacking.

### 5.4 Indirect cellular mechanisms derived from AD research

Several pathways discussed in the ApoE4 literature may affect neuronal resilience to injury, yet direct validation in CSVD is currently lacking. These mechanisms are not mutually exclusive; rather, they may collectively reflect a reduced capacity of neurons to adapt to chronic hypoperfusion and metabolic stress in the context of ApoE4: abnormalities in energy metabolism can increase endoplasmic reticulum and mitochondrial loads, while impairments in the endosomal-lysosomal system and autophagy may limit membrane lipid turnover and synaptic homeostasis. Evidence supporting ApoE4-associated neuronal insulin receptor retention in endosomes, altered PI3K-Akt signaling, and mitochondrial energy stress stems primarily from AD-related models or targeted replacement models (Safieh et al., 2024; Zhao et al., 2017; Chen et al., 2025; Budny et al., 2025; Nyberg et al., 2025). Since CSVD itself causes chronic hypoperfusion and metabolic stress, the concept of “impaired energy adaptability” is biologically plausible; however, an ApoE4-specific mechanism involving insulin receptor trafficking has not yet been proven to drive CSVD-CI. These cellular pathways, supported primarily by indirect evidence, are summarized in Figure 4.

**Figure 4:**
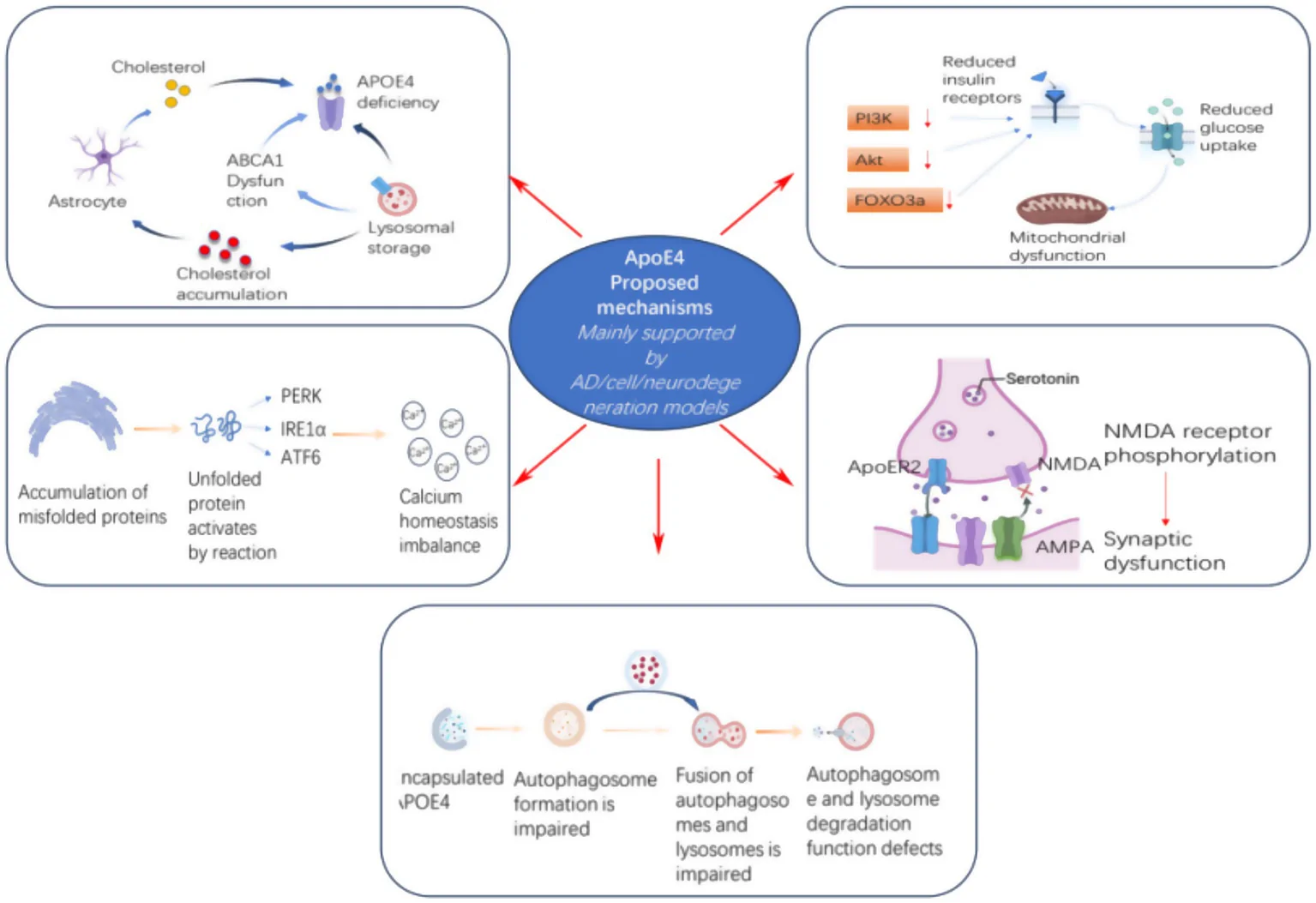
Predominantly indirect molecular pathways proposed to link ApoE4 with impaired brain metabolic homeostasis and neuronal resilience. The illustrated pathways include cholesterol/lipid trafficking, insulin and energy metabolism, endoplasmic reticulum stress, synaptic signaling, and autophagy–lysosomal function. These mechanisms are supported mainly by Alzheimer’s disease, cell, or other neurodegenerative models and should not be interpreted as established mechanisms of sporadic CSVD.

Similarly, ApoE4-associated endoplasmic reticulum (ER) stress and the unfolded protein response have been observed mainly in cellular and neurodegenerative disease models (Steele et al., 2022; Martens et al., 2022). Chronic ischemia can itself induce ER stress (Han et al., 2021), suggesting a potential point of convergence; however, there is currently no evidence establishing the existence of ApoE4-specific ER stress pathways in human CSVD. Consequently, these findings should serve as a basis for generating research hypotheses rather than being characterized as disease-specific mechanisms (Steele et al., 2022; Han et al., 2021).

Effects related to synaptic function and receptor trafficking—including Reelin/ApoER2 signaling—have also been demonstrated primarily in the contexts of neurons and Alzheimer’s disease (Safieh et al., 2024; Yi et al., 2024; Yao et al., 2026). While synaptic dysfunction is indeed a common final pathway for cognitive impairment, attributing CSVD-CI to Reelin–Dab1 or related signaling requires direct evidence that vascular injury can activate this pathway in ApoE4 carriers. Similar caution applies to autophagy-lysosome dysfunction: although ApoE4 can impair endolysosomal and autophagic homeostasis in neurodegenerative disease models (Asiamah et al., 2024; Belaidi et al., 2025), its causal role in CSVD remains unproven. These indirect mechanisms are discussed only briefly here, as elaborating on them beyond the available evidence could obscure vascular and lipid pathways that are more specific to CSVD (Windham and Cohen, 2024; Ter Telgte et al., 2018).

Taken together, the strongest mechanistic link between ApoE4 and CSVD is not a single molecular cascade. It is more likely that, in the presence of vascular stress, ApoE4 reduces the resilience of cerebral vessels and white matter to injury by impairing neurovascular regulation and altering lipid processing. There is robust evidence supporting BBB dysfunction in CSVD, and significant support for ApoE4-associated neurovascular vulnerability in CSVD-like models; meanwhile, microglial lipid droplets represent a compelling yet unverified hypothesis specific to CSVD. In contrast, mechanisms such as CypA–MMP-9, TREM2–ApoE, insulin receptor retention, Reelin-related signaling, endoplasmic reticulum stress, and autophagy should be clearly categorized as indirect evidence until they are replicated in CSVD patient populations or models. Table 1 details the levels of evidence for each ApoE4–CSVD mechanism.

## 6 Future therapeutic directions

Currently, no ApoE4-targeted intervention has been proven effective for CSVD-CI; existing evidence stems primarily from studies on Alzheimer’s disease (AD), traumatic brain injury, cell cultures, and other preclinical systems. Consequently, ApoE-targeted strategies should be viewed as avenues for research rather than immediate therapeutic options for CSVD. Standardized management of modifiable vascular risk factors—particularly hypertension, diabetes, smoking, dyslipidemia, physical inactivity, and other drivers of systemic vascular injury—remains more directly aligned with established biological mechanisms of CSVD.

Potential ApoE-targeted platforms include ApoE4 structural correction, ApoE-mimetic peptides, enhancement of ABCA1-dependent lipidation, modulation of receptor trafficking, and the reduction or immune-targeting of ApoE4-associated pathogenic effects (Jackson et al., 2024; Yassine et al., 2025; Zhang et al., 2026; Poblano et al., 2024). For CSVD, these strategies must first establish a mechanistic link to vascular outcomes. A candidate intervention should first demonstrate, in ApoE4-CSVD models, an ability to improve cerebral blood flow regulation, endothelial or pericyte function, BBB integrity, white matter perfusion, myelin preservation, or white matter hyperintensity (WMH)-like pathology. Merely reducing a specific AD molecular biomarker does not constitute sufficient evidence. Table 3 briefly compares these exploratory strategies, the primary model systems providing the evidence, and their current relationship to CSVD. Particular caution is warranted regarding immunotherapies or strategies aimed at lowering ApoE levels, given ApoE’s physiological roles in lipid transport, tissue repair, and immunomodulation. Excessive neutralization of ApoE could compromise beneficial lipid supply or adaptive responses; therefore, vascular safety must be assessed prior to translational application. Similarly, therapies targeting lipid droplets or microglial metabolism should distinguish between pathological lipid accumulation and protective lipid sequestration (Windham and Cohen, 2024; Lee et al., 2023; Yassine et al., 2025).

**Table 3:** Potential ApoE4-targeted therapeutic directions and current evidence in CSVD-CI.

Therapeutic strategy	Proposed mechanism	Representative approaches	Disease model/current evidence	CSVD relevance and limitation
ApoE4 structure correction	Restore ApoE4 abnormal conformation by disrupting ApoE4-specific domain interaction (Arg61-Glu255), thereby reducing conformational toxicity and improving cellular function	GIND25; PH002	Preclinical studies indicate that structure correctors can partially convert ApoE4 toward an ApoE3-like conformation and improve neuronal morphology and mitochondrial dysfunction in experimental models	Evidence is mainly derived from AD-related models; limited data in CSVD-CI; long-term safety and clinical efficacy remain unclear
ApoE mimetic peptides	Mimic ApoE lipid-binding and receptor-interacting functions to improve lipid transport and regulate inflammatory responses	Ac-hE18A-NH₂; CN-105; COG112; COG1410	Experimental studies suggest potential neuroprotective and anti-inflammatory effects, mainly in AD and inflammatory disease models	Isoform-specific effects remain uncertain; direct evidence in CSVD-CI is lacking
Enhancement of ApoE lipidation	Increase ABCA1/ABCG1-mediated cholesterol efflux and ApoE lipidation through LXR/RXR pathway activation, improving ApoE4-associated lipid transport dysfunction	LXR/RXR agonists; Bexarotene; CS-6253	Animal studies suggest enhanced ApoE lipidation and improved cognitive phenotypes in ApoE4 models	Systemic metabolic effects limit clinical application; efficacy in human CSVD remains unverified
Regulation of ApoE receptors	Restore LDL receptor family-mediated signaling, including ApoER2, LDLR, and LRP1 pathways, to improve synaptic function and neuronal homeostasis	ApoER2 restoration; receptor trafficking modulation	ApoE4 has been shown to impair ApoER2 expression and recycling, affecting reelin-dependent synaptic signaling in experimental models	Receptor-specific mechanisms in CSVD-CI remain poorly defined; therapeutic approaches are still at an early stage
Immunotherapy targeting ApoE4	Reduce pathological ApoE4 accumulation or modulate ApoE-associated neuroinflammatory responses	Anti-ApoE antibodies (HJ6.3, HAE-4); ApoE-targeting ASOs/siRNA	AD models suggest that ApoE reduction strategies may decrease pathological changes and improve cognitive performance	Most studies focus on amyloid-related pathology; potential effects on vascular dysfunction and CSVD-CI require validation
Lifestyle and metabolic intervention	Improve vascular metabolic status, lipid homeostasis, and inflammatory environment to reduce ApoE4-associated cognitive vulnerability	Mediterranean diet; omega-3 fatty acids; dietary metabolic regulation; physical activity	Epidemiological and clinical studies suggest possible cognitive benefits in ApoE4 carriers through vascular and metabolic improvements	Effects are inconsistent across studies; optimal intervention strategies for ApoE4 carriers remain undefined

No listed approach has established efficacy in CSVD-CI; disease models and translational limitations are stated explicitly.

Preclinical development should utilize aged ApoE4 and ApoE3 models and incorporate chronic hypoperfusion, hypertension, diabetes, or other vascular comorbidities, while pre-specifying outcome measures related to MRI, the BBB, small-vessel histopathology, white matter integrity, and cognition. Human translational studies should begin with genotype-stratified cohorts of patients with cerebral small vessel disease (CSVD) for whom extensive biomarker data are available, rather than simply extrapolating efficacy findings from Alzheimer’s disease (AD). Until these steps are completed, any conclusions regarding such therapies should be explicitly characterized as exploratory (Montagne et al., 2020; Windham and Cohen, 2024; Ter Telgte et al., 2018; Montagne et al., 2020; Markus and de Leeuw, 2023).

## 7 Research priorities and conclusions

Current research needs to focus on CSVD itself rather than simply extrapolating mechanisms from Alzheimer’s disease (AD). Priority human studies should include prospective cohorts with APOE genotyping that utilize MRI markers meeting STRIVE standards to quantitatively assess white matter hyperintensity (WMH) progression, BBB permeability, cerebral perfusion, and cognitive trajectories, while rigorously adjusting for age, sex, ancestry, hypertension, diabetes, lipid status, renal function, overall vascular risk, APOE gene dosage, and AD biomarkers. Cerebral amyloid angiopathy (CAA) should be classified separately or analyzed as a pre-specified subgroup.

The “brain–heart–kidney axis hypothesis” should be tested by integrating brain MRI with measurements of cardiac and renal microvasculopathy, rather than being accepted merely based on shared risk factors (Ter Telgte et al., 2018). The microglial lipid droplet hypothesis requires direct investigation of white matter in human CSVD using lipidomics, cell-type-resolved transcriptomics, histopathology, and spatial omics, focusing on ACSL1, ApoE genotype, lipid droplet composition, BBB leakage, oligodendrocyte injury, and lesion stage (Lee et al., 2023; Marschallinger et al., 2020). Longitudinal studies are particularly crucial to determine whether lipid imbalance precedes WMH expansion or represents a secondary response.

In summary, existing evidence favors viewing ApoE4 as a modifier of cerebrovascular susceptibility rather than a direct causative gene for sporadic CSVD. Population-based epidemiological associations show significant heterogeneity; CAA represents a distinct amyloid-related context and should not be extrapolated to hypertensive CSVD. Direct CSVD research strongly supports endothelial dysfunction, BBB disruption, and white matter injury as key pathological processes, while the concept of ApoE4-specific neurovascular vulnerability is supported by vascular stress models. Recently proposed hypotheses regarding systemic small vessel disease and microglial lipid droplets have clarified testable questions, yet they remain hypotheses. Only by strictly distinguishing direct evidence of CSVD from mechanistic inferences derived from AD can we determine whether ApoE4 might serve as a reliable biomarker or therapeutic target for CSVD-CI.
